# Trapping of Organochlorine Compounds in High Mountain Lakes

**DOI:** 10.1100/tsw.2001.320

**Published:** 2001-10-30

**Authors:** Joan O. Grimalt, Pilar Fernandez, Rosa M. Vilanova

**Affiliations:** Department of Environmental Chemistry, I.C.E.R-C.S.I.C, Catalonia, Spain

**Keywords:** persistent organic pollutants, snow, high mountain lakes, temperature effects

## Abstract

High mountain areas have recently been observed to be polluted by organochlorine compounds (OC) despite their isolation. These persistent pollutants arrive at these remote regions through atmospheric transport. However, the mechanisms involving the accumulation of these compounds from the atmospheric pool to the lacustrine systems still need to be elucidated. These mechanisms must be related to the processes involving the transfer of these pollutant from low to high latitudes[1] as described in the global distillation effect[2].

A recent study by Grimalt et al., published in the first July issue of *Environmental Science and Technology*, outlines the importance of the temperature effects for the accumulation of OC[3]. The study was based on the analysis of these compounds in fish and sediments from 19 European high mountain lakes (40–67ºN). Fish concentrations and sediment inventories showed that an OC fraction, involving the less volatile compounds (sub-cooled liquid vapor pressure $P_L \leq 10^{-2.5}$ Pa), was retained to a major extent in the locations situated at higher altitude, those of lower annual average air temperature. This general trend was independent of OC source and was not significantly influenced by possible local polluted sites. The phase-change pseudo enthalpies calculated from the sediment inventories closely matched the summed theoretical volatilization and dissolution enthalpies. In the case of fish, the pseudo-enthalpies were even higher, pointing to an amplification mechanism.

The results are consistent with previous studies describing the latitudinal OC distribution[1,4]. Areas of high accumulation of the more volatile compounds such as hexachlorobenzene (HCB; $P_L = 10^{-1.2}$) are located at 60 and 70ºN, with mean temperatures between –7 and –12ºC[5]. The average air temperatures of the studied lakes above 2400 m ranged in the range of –2 and 1ºC which is insufficient for trapping volatile compounds such as HCB but allows the retention of the less volatile compounds. These, in turn, are mobilized at sea level mean temperatures such as 5–10ºC[6].

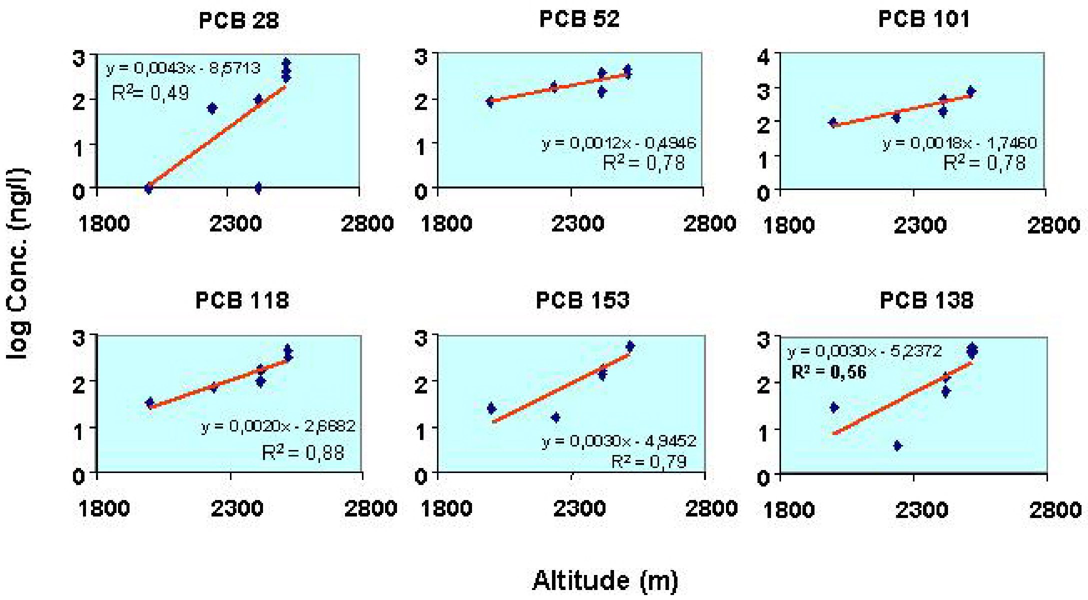

**Figure 1.** Concentrations of polychlorobiphenyl congeners in snow of high altitude lakes.

Further understanding into these temperature effects was obtained from the analysis of OC in the snow accumulated in these lakes during the cold periods. Snow cores were collected and analyzed for OC in a subset of the lakes considered in Grimalt et al.[3]. The concentrations and deposition fluxes are described in Carrera et al.[7]. Only polychlorobiphenyls (PCBs) and HCB were found above method detection limit in most samples. The snow PCB congener distributions changed significantly between sites but in general a predominance of the less chlorinated congeners was observed.

Representation of the data vs. altitude showed again higher concentrations as higher is the location of the lake sites (Fig. 1). The trend was observed for all OC, not only for the less volatile although the samples corresponding to the highest lakes were those exhibiting higher relative proportion of the more chlorinated congeners[7]. Comparison of these PCB mixtures with those in fish and sediments suggests that the more volatile compounds trapped in snow deposition may be subsequently transferred to the atmosphere in the warm periods, e.g., during snow melting, and that only a fraction of the less volatile PCBs will be retained in the lake. The extension of this fraction will depend on the temperatures at each site.
